# Cases of Peritoneal Inflammation Succeeded by Phlegmatia Dolens

**Published:** 1818-05

**Authors:** G. Williams

**Affiliations:** Surgeon


### *Cases of Peritoneal Inflammation succeeded by Phlegmatia Dolens.* By G. Williams, Surgeon.

A Delicate woman, the third day after an easy labour with her third child, was attacked with peritonæal inflammation. The spirits were considerably depressed; the sense of debility extreme. Pressure on the abdomen, especially just above the pubis, caused most acute pain. Pulse quick, small, and hard. Tongue white; face flushed; bowels constipated; lochia suppressed; secretion of milk stopped.

The abstraction of sixteen ounces of blood, bearing strong marks of inflammation, was immediately followed by a cathartic of jalap and calomel, and a dose of a strong solution of Epsom salts in infusum sennæ every two hours. Fomentations to the abdomen.

The motions procured by the cathartics were of the colour of pitch, and dreadfully offensive.

By the sixth day these symptoms had disappeared; but the patient complained of a violent pain in the calf of the right leg, which resembled the cramp, and came on suddenly in the night. An excessively tender lump, the size of a hazel-nut, consisting of several smaller ones, could be felt beneath the integuments at the site of the pain. The leg was slightly swelled.

On the next day, febrile symptoms appeared, and the limb swelled from the groin to the toes, the whole of it being so exquisitely sensible that the weight of the bed-clothes could not be borne. Its surface was smooth and shining, as white as a sheet of writing-paper, and did not pit on pressure. On rubbing the finger along, it felt knotty: these knots varied in size, from a small pea to a French bean, and were most numerous in the course of the principal trunks of the absorbents.

The treatment consisted in the application of leeches to the inguinal region, with fomentations; and after a few days, when the morbid sensibility of the limb had diminished, camphorated embrocations.

When the limb had nearly regained its natural size, another accession of fever took place. The feet were stone cold; the head and face hot; the temporal arteries throbbed violently; the eyes inflamed and irritable; the patient so delirious as to be with difficulty confined to bed. Cloths wet in cold vinegar and water were constantly applied to the shaved head; a blister put to the nucha, the feet immersed in warm water, and a large dose of calomel administered, followed by a cathartic mixture. By keeping up a constant and brisk action of the bowels for some days, the woman became convalescent, and ultimately recovered.

Mary Newberry, of a florid complexion and full habit, had an attack of peritonitis on the third or fourth day after delivery of her first child. The labour was easy. The child had been dead two or three days. One copious bleeding, with purging and fomentations, removed all tenderness from the abdomen, re-established the lochial discharge, and diminished the febrile symptoms. On the eighth day, the right leg was affected with phlegmatia dolens: its attack was sudden, as in the preceding case; the treatment was the same. The swelling continued undiminished for near a fortnight. A bilious vomiting and diarrhœa now appeared, the leg subsided rapidly, and the patient recovered.

The above Cases are illustrative of the metastasis of morbid action, or increased momentum, from one part of the system to another. It will be seen, that in both the transition was from the peritonæum to the cellular membrane of a lower extremity; from thence, in the first, to the cranial contents; in the second, to the mucous membrane of the intestines.

I am not aware of any dissections of phlegmatia dolens on record; we may therefore conclude, that though a most distressing and painful affection, it does not often prove fatal. A case fell under my observation in the early part of my professional studies, respecting which my memory now furnishes me with only the following particulars:—The woman was a very delicate subject, had an easy labour, but flooded alarmingly after. She was delirious when I saw her, but that was attributed to "excessive debility," and treated with nourishing diet, cordials, and stimulants. The patient died. No dissection was made. Here it is evident the patient perished of determination to the brain, not the affection of the lower extremity.

It may be worthy of remark, that in the two first instances related above, the attack of phlegmatia dolens was preceded by peritonitis and depletion; and in the third, by "alarming" uterine hæmorrhage.

G. WILLIAMS.

*Portsea, April 9, 1818.*

